# P-787. Descriptive Analysis of Treatment Failure in Uncomplicated Urinary Tract Infection Among US Female Patients with Diabetes: Stratified by Level of Diabetes Control

**DOI:** 10.1093/ofid/ofaf695.998

**Published:** 2026-01-11

**Authors:** Pamela Kushner, Seth Kuranz, Virginia Noxon-Wood, Meghan E Luck, Darrian Tattoli, Tien-Huei Hsu, Jeffrey J Ellis

**Affiliations:** University of California Irvine Medical Center, Orange, CA, United States, Orange, California; Inovalon, Bowie, MD, United States, Bowie, Maryland; Inovalon, Bowie, MD, United States, Bowie, Maryland; GSK, Brattleboro, VT; GSK, Collegeville, PA, United States, Collegeville, Pennsylvania; GSK, Collegeville, PA, United States, Collegeville, Pennsylvania; GSK, Brattleboro, VT

## Abstract

**Background:**

Patients (pts) with uncontrolled diabetes mellitus (DM) pose a greater challenge in the treatment of urinary tract infections (UTIs) than pts with controlled DM. We compared treatment failure (TF) rates in female pts with varying DM control, who were treated for otherwise uncomplicated UTI (uUTI).

**Figure:**
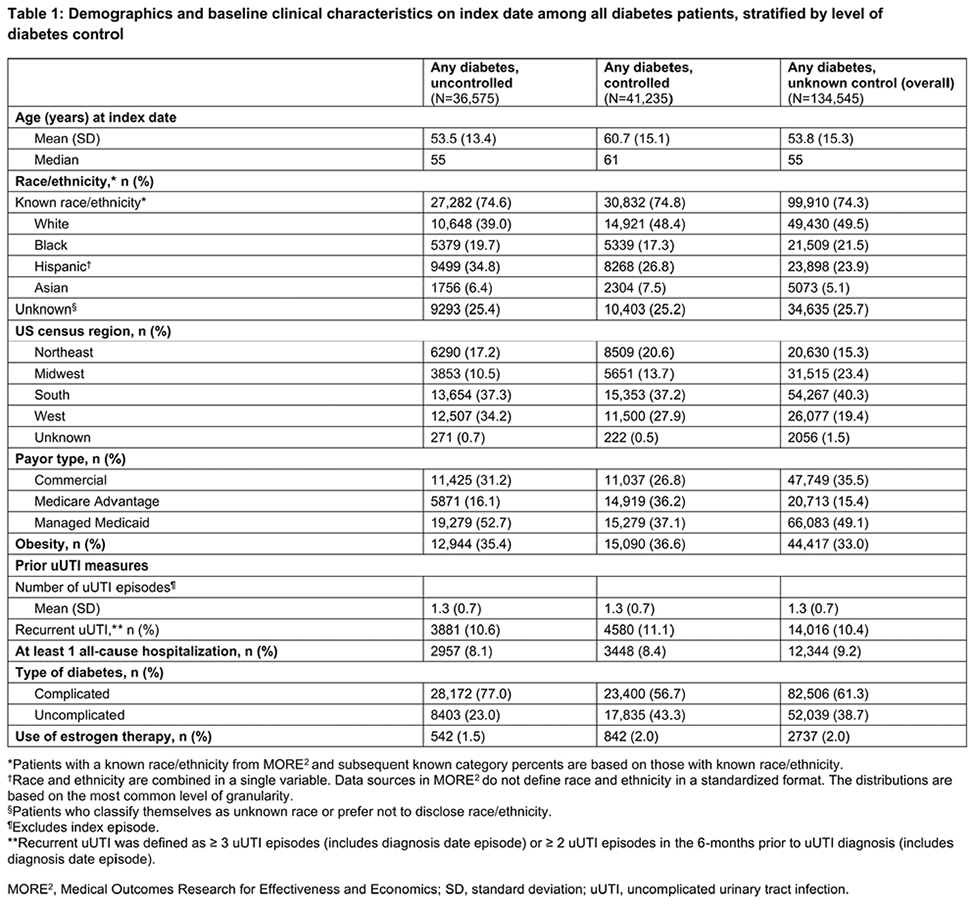

**Figure:**
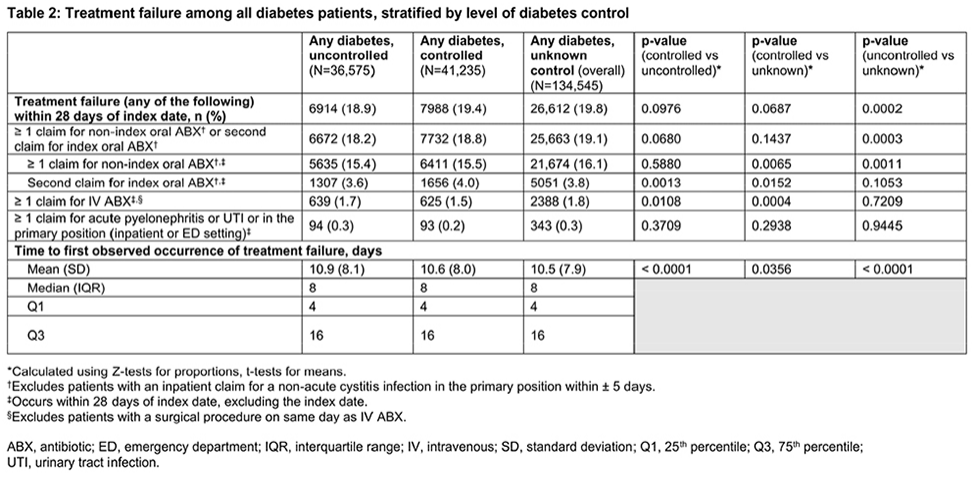

**Methods:**

This observational, retrospective analysis of the real-world deidentified Inovalon MORE^2^ Registry included female pts aged ≥ 12 years with a uUTI diagnosis (Dx) between Jan 2018 and Dec 2022. Index date was the first oral antibiotic (ABX) claim observed ± 5 days of uUTI Dx. Pts were included if either of the following was observed in the 12 months prior to the index date: ≥ 1 claim with a Dx of DM and ≥ 1 claim for an antidiabetic treatment, or ≥ 2 claims with a Dx of DM 1–365 days apart. DM control was based on pts’ age (years) and 12-month pre-index glycosylated hemoglobin (HbA1c [mmol/mol]): controlled DM = age < 65 with a HbA1c < 7 or age ≥ 65 with a HbA1c < 8; uncontrolled DM = age < 65 with a HbA1c ≥ 7 or age ≥ 65 and HbA1c ≥ 8; and, unknown control = pts without a HbA1c. TF was defined as having a second oral ABX, intravenous ABX, or emergency department or inpatient stay with a primary Dx of UTI ≤ 28 days after the index date. Pairwise comparisons of TF between control groups were made using Z-tests for proportions and t-tests for means.

**Results:**

Overall, 212,355 female uUTI pts with DM were identified: uncontrolled DM, 36,575 (17.2%); controlled DM, 41,235 (19.4%); unknown control, 134,545 (63.4%). Pts with controlled DM were older while the uncontrolled group was comprised of greater percentages of minority and managed Medicaid pts (Table 1). TF occurred in 18.9%, 19.4%, and 19.8% of the uncontrolled, controlled, and unknown control groups, respectively (p=0.0976, unadjusted comparison: uncontrolled vs controlled; Table 2). Mean time to first observed TF ranged from 10.5 to 10.9 days (median = 8, all groups).

**Conclusion:**

This descriptive analysis found high TF in female uUTI pts with DM; however, minimal differences were seen in TF rates across levels of DM control. Given the imbalance observed in various baseline characteristics and the majority of study pts having unknown control status, further research is warranted to ascertain the independent impact of DM control.

Funding: GSK study 222865.

**Disclosures:**

Pamela Kushner, MD, GSK: Advisor/Consultant|GSK: Speaker Seth Kuranz, PhD, Inovalon: Employee of Inovalon, a consulting company that received funding from GSK to conduct this study. Virginia Noxon-Wood, PhD, Inovalon: Employee of Inovalon, a consulting company that received funding from GSK to conduct this study. Meghan E. Luck, PharmD, GSK: Employee|GSK: Stocks/Bonds (Public Company) Darrian Tattoli, PharmD, GSK: Employee|GSK: Stocks/Bonds (Public Company) Tien-Huei Hsu, PhD, GSK: Employee|GSK: Stocks/Bonds (Public Company) Jeffrey J. Ellis, PharmD, MS, GSK: Employee|GSK: Stocks/Bonds (Public Company)

